# Doxycycline-Doped Polymeric Membranes Induced Growth, Differentiation and Expression of Antigenic Phenotype Markers of Osteoblasts

**DOI:** 10.3390/polym13071063

**Published:** 2021-03-28

**Authors:** Manuel Toledano-Osorio, Francisco J. Manzano-Moreno, Manuel Toledano, Antonio L. Medina-Castillo, Victor J. Costela-Ruiz, Concepción Ruiz, Raquel Osorio

**Affiliations:** 1 Colegio Máximo de Cartuja s/n, Faculty of Dentistry, University of Granada, 18071 Granada, Spain; mtoledano@correo.ugr.es (M.T.-O.); rosorio@ugr.es (R.O.); 2 Medicina Clínica y Salud Pública Programme, University of Granada, 18071 Granada, Spain; 3 Biomedical Group (BIO277), Department of Stomatology, School of Dentistry, University of Granada, 18071 Granada, Spain; fjmanza@ugr.es; 4 Instituto Investigación Biosanitaria, ibs.Granada, 18071 Granada, Spain; vircoss@gmail.com (V.J.C.-R.); crr@ugr.es (C.R.); 5 NanoMyP^®^, Nanomateriales y Polimeros S.L., Spin-Off Company of the University of Granada, Edificio BIC-Granada, Av. Innovación 1, Armilla, 18016 Granada, Spain; amedina@nanomyp.com; 6 Biomedical Group (BIO277), Department of Nursing, Faculty of Health Sciences, University of Granada, 18071 Granada, Spain; 7 Institute of Neuroscience, University of Granada, Centro de Investigación Biomédica (CIBM), Parque de Tecnológico de la Salud (PTS), 18071 Granada, Spain

**Keywords:** CD markers, doxycycline, antigenic phenotype, osteoblasts, membrane

## Abstract

Polymeric membranes are employed in guided bone regeneration (GBR) as physical barriers to facilitate bone in-growth. A bioactive and biomimetic membrane with the ability to participate in the healing and regeneration of the bone is necessary. The aim of the present study was to analyze how novel silicon dioxide composite membranes functionalized with zinc or doxycycline can modulate the osteoblasts’ proliferation, differentiation, and expression of selected antigenic markers related to immunomodulation. Nanostructured acrylate-based membranes were developed, blended with silica, and functionalized with zinc or doxycycline. They were subjected to MG63 osteoblast-like cells culturing. Proliferation was assessed by MTT-assay, differentiation by evaluating the alkaline phosphatase activity by a spectrophotometric method and antigenic phenotype was assessed by flow cytometry for selected markers. Mean comparisons were conducted by one-way ANOVA and Tukey tests (*p* < 0.05). The blending of silica nanoparticles in the tested non-resorbable polymeric scaffold improved the proliferation and differentiation of osteoblasts, but doxycycline doped scaffolds attained the best results. Osteoblasts cultured on doxycycline functionalized membranes presented higher expression of CD54, CD80, CD86, and HLA-DR, indicating a beneficial immunomodulation activity. Doxycycline doped membranes may be a potential candidate for use in GBR procedures in several challenging pathologies, including periodontal disease.

## 1. Introduction

Guided bone regeneration (GBR) is a frequently used surgical technique. GBR techniques are intended to obtain enough alveolar bone volume to accomplish a successful therapy with osseointegrated dental implants. In order to achieve this goal, the use of a membrane, that should not only act as a physical barrier but also as a modulatory barrier, protecting the growth of osteoblastic cells is required [1]. GBR is a tissue engineering therapy that greatly relies on the appropriate selection of the biomaterial. Commercially available membranes can be resorbable or non-resorbable [2]. The shortcoming for resorbable membranes is mainly a lack of space maintenance due to short resorption times. On the other hand, for non-resorbable membranes, higher rates of wound dehiscences and the need for a second surgery in order to retrieve the membrane are the main drawbacks, as these kinds of membranes do not integrate with the host bone tissue [3]. 

The search for new osteogenic, bioactive, and biomimetic non-resorbable membranes that integrate with the bone tissue structure is crucial. The electrospinning technique can be used for obtaining scaffolds with attractive features, such as the similarity to the extracellular matrix [4], and may also be successfully used for drug delivery and tissue regeneration [5,6]. Recently, a new membrane barrier based on the electrospun of a polymer mixture of (MMA)_1_-co-(HEMA)_1_ and (MA)_3_-co-(HEA)_2_ has been developed. These nanofibrous scaffolds closely mimic the bone collagen matrix (fibers with diameters ranging from 50 to 500 nm) [7]. The composite membranes were formed with silica nanoparticles and functionalized with zinc or doxycycline. These new composite membranes combine the mechanical properties of polymeric materials, the bioactivity of SiO_2_-NPs [7], osteogenicity provided by the doped zinc [8], and antibacterial properties conferred by the doxycycline [9]. Moreover, zinc has been tested in vivo and in vitro studies, concluding that it plays a crucial role in inducing the viability and proliferation of bone cells as well as enhancing new bone formation and mineralization of extracellular matrix [8,10,11]. Similarly, doxycycline appears to enhance maturation and cells’ osteogenic capacity [7,12,13]. 

These novel membranes should also be tested on the cell’s environment. It would be desirable that the membranes favored osteoblast proliferation and differentiation. It may be positive that the membranes could contribute to a positive immunomodulation, hindering the establishment of a pro-inflammatory atmosphere and hampering the penetration of pro-inflammatory cells and cytokines, thus improving tissue regeneration [14]. A growing body of evidence has been found about the immunoregulatory potential of biomaterials. It has been already shown that interactions of osteoblasts with biomaterials can regulate the extent of the response initiated by macrophages, mainly by osteoblastic release of interleukin-6, prostaglandin-E2, or granulocyte macrophage colony-stimulating factor [15,16]. The bone and immune systems are closely related through cellular and molecular interactions [17]. Several regulatory molecules are shared by the immune and skeletal systems, which include cytokines, receptors, signaling molecules, and transcription factors [17,18]. 

The cluster of differentiation (CD) is a protocol used for the identification of cells’ surface antigens, providing targets for immunophenotyping of cells. In terms of physiology, CD molecules can act in numerous ways, often acting as important receptors or ligands to the cell. The behavior of the cell is usually modified by CD proteins playing a role in cell signaling or other functions, such as cell adhesion. Assessing CD osteoblasts’ antigenic phenotype is possible by means of flow cytometry. It is an extremely robust technique that has been successfully implemented to probe phenotypic cellular activity in living cells. Namely, the in vitro immunomodulatory properties of bone cells may be ascertained by determining the deficiency or overexpression of the related CD antigenic markers of interest at a specific time point of cells’ differentiation stages. It may provide information about cells’ potential role in immunomodulation [19].

The aim of the present study was to analyze the proliferation, differentiation potential, and immunomodulation ability of osteoblastic cells cultured on silicon dioxide composite membranes functionalized with zinc or doxycycline. The null hypothesis is that zinc or doxycycline addition on membranes does not affect the proliferation, differentiation, and antigenic phenotype expression of the cultured osteoblasts.

## 2. Materials and Methods

### 2.1. Preparation of Nanostructured Polymeric Membranes

Nanostructured membranes were produced by electrospinning using a novel polymeric blend: (MMA)_1_-co-(HEMA)_1_/(MA)_3_-co-(HEA)_2_ 50/50 wt.%, doped with 5 wt.% of SiO_2_-NPs (NanomyP^®^, Granada, Spain). The membranes were incubated for 2 h in a sodium carbonate buffer solution (333 mM; pH = 12.5), so as to activate them with carboxyl groups (HOOC-Si-M). The partial hydrolysis of ester bonds and the disposal of carboxyl groups on the surface of the artificial tissue made this functionalization possible [20]. The membranes were then rinsed with distilled water and dried using a vacuum oven [21]. Secondly, the membranes were functionalized with zinc or doxycycline (Dox). Zinc was incorporated using the ability of carboxyl groups to complex divalent cations. On the other hand, Dox was immobilized on the membranes by acid–base reactions between amino groups of Dox and carboxyl groups present in the membranes. HOOC-Si-M were immersed under continuous shaking at room temperature and in aqueous solutions (pH = 7) of both 330 mgL^−1^ of ZnCl_2_ or 800 mgL^−1^ of Dox [7]. Four different membranes were tested: (1) non-functionalized and SiO_2_-NPs undoped membrane (HOOC-M), (2) SiO_2_-NPs doped membrane (HOOC-Si-M), (3) SiO_2_-NPs doped membrane functionalized with Zn (Zn-HOOC-Si-M), and (4) SiO_2_-NPs doped membrane functionalized with Dox (Dox-HOOC-Si-M). The membranes were placed at the bottom of a 24-well plate (Falcon, Becton Dickinson Labware, Franklin Lakes, NJ, USA) and sterilized using an ultraviolet radiation sterilization desk (J.P. SELECTA, Barcelona, Spain).

### 2.2. Cell Culture

The human MG63 osteosarcoma cell line was purchased from the American Type Culture Collection (ATCC, Manassas, VA, USA). This cell line has been widely used as an osteoblast model since it shares most of the same characteristics with primary human osteoblasts. MG63 osteoblast-like cells have no interspecies differences with primary human osteoblasts, they have a shorter isolation time and there is unlimited accessibility [1]. The MG63 cell line was maintained in Dulbecco’s modified Eagle medium (DMEM; Invitrogen Gibco Cell Culture Products, Carlsbad, CA, USA) with penicillin 100 IU/mL (Lab Roger SA, Barcelona, Spain), gentamicin 50 mg/mL (Braum Medical SA, Jaen, Spain), amphotericin B 2.5 mg/mL (Sigma, St. Louis, MO, USA), 1% glutamine (Sigma), and 2% HEPES (Sigma) supplemented with 10% of fetal bovine serum (FBS; Gibco, Paisley, UK), as described by Díaz Rodríguez et al. [22]. Cultures were kept in a humidified atmosphere at 37 °C of 95% air and 5% CO_2_. Cells were detached from the flask using 0.05% trypsin (Sigma) and 0.02% ethylenediaminetetraacetic acid (EDTA; Sigma) solution. After this process, they were rinsed and resuspended in complete culture medium with 10% FBS as described by Manzano-Moreno et al. [19]. 

### 2.3. Cell Proliferation Assay 

Osteoblasts were seeded at 1 × 10^4^ cells/mL per well onto the membranes placed inside the 24-well plate. The cells were then cultured in a humid atmosphere of 95% air and 5% CO_2_ at 37 C for 48 h. After this time, cell proliferation was assessed by means of the 3-(4,5-dimethylthiazol-2-yl)-2,5-diphenyltetrazolium (MTT) assay. For this purpose, the media was replaced by phenol red-free DNEM with MTT 0.5 mg/mL (Sigma) and incubated for 4 h. MTT cellular reduction resulted in the formation of insoluble crystal deposits of formazan that were dissolved by adding dimethyl sulfoxide (Merck Biosciences, Darmstadt, Germany). Absorbance was then measured with a spectrophotometer (Sunrise, Tecan, Männedorf, Switzerland) at 570 nm [23]. The results were expressed as mean absorbance ± standard deviation (SD). At least three experiments were conducted for each type of membrane. 

### 2.4. Field Emission Scanning Electron Microscopy (FESEM)

Osteoblasts were also seeded at 1 × 10^4^ cells/mL per well onto the membranes placed inside the 24-well plate. The cells were then cultured in a humid atmosphere of 95% air and 5% CO_2_ at 37 °C for 48 h. After this time, two membranes of each experimental group were submitted to FESEM (GEMINI, Carl Zeiss SMT, Oberkochen, Germany) observation. Samples were first critical point dried and covered with carbon. 

### 2.5. Alkaline Phosphatase Activity 

Early osteoblasts differentiation was assessed by evaluating the alkaline phosphatase (ALP) activity, a colorimetric assay (Diagnostic kit 104-LL, Sigma). This method, measures the conversion of the colorless substrate p-nitrophenyl phosphate to the yellow p-nitrophenol, accomplished by the ALP enzyme. The rate of color shift corresponded with the amount of enzyme present in the culture. Standards of p nitrophenol (0–250 μM) were prepared from dilutions of a 1000 μM stock solution and assayed in parallel. The ALP assay was performed as previously described by Manzano-Moreno et al. [23]. In brief, cell cultures, for 72 h on the membranes placed inside the 24-well plate, were trypsinized (0.05% trypsin, 1 mM EDTA, (Invitrogen Gibco)) and lysed in 100 μL 1 M Tris pH 8.00 by ultrasonification for 4 min. Then, the suspension was mixed with a 7.6 mM p-nitrophenyl phosphate solution at a proportion of 1:10 and incubated for 15 min at 37 °C. Substrate solution was prepared by merging an aqueous solution of 4 mg/mL of 4-nitrophenyl phosphate disodium salt (Sigma) with an equal volume of 1.5 M alkaline buffer (Sigma). The reaction was stopped by adding 1 mL 0.05 N NaOH, and the final absorbance was measured with a spectrophotometer (Sunrise, Tecan, Männedorf, Switzerland) at 405 nm. The total protein content was estimated by the Bradford method using a protein assay kit from Bio-Rad Laboratories (Bio-Rad Laboratories, Nazareth-Eke, Belgium). All samples were conducted in triplicate.

### 2.6. Antigenic Phenotype 

Antigenic phenotype was assessed by flow cytometry at 48 h of culture using 0.05% trypsin (Sigma) and 0.02% EDTA (Sigma) solution 0.4% EDTA solution. They were then washed and suspended in PBS at a concentration of 2 × 10^4^ cells/mL. Osteoblasts were labeled by direct staining with anti-CD54, CD80, CD86, and HLA-DR monoclonal antibodies (MAbs); CD54/IOL1b, CD80, CD86, and OKDR, respectively (Invitrogen Corp). 100 μL of cell suspension were incubated with 10 μL of each MAb for 30 min at 4 °C in the absence of light. Cells were rinsed and re-suspended in PBS 1 mL, and analyzed at a wavelength of 488 nm in a flow cytometer with a diode laser (FACSCanton II, Becton-Dickinson, Palo Alto, CA, USA) to determine the percentage of fluorescent cells [24].

### 2.7. Phagocytic Activity

Phagocytic activity was also studied by flow cytometry following the method described by Díaz-Rodriguez et al. [25]. In brief, cultured human MG-63 osteosarcoma cells were cultured for 48 h on the tested membranes. After this time, they were detached from the membranes, washed, and then suspended in complete culture medium with 10% FBS at a density of 2 × 10^4^ cells/mL. Cells were treated by direct staining with labeled latex beads; a 100-µL cell suspension was incubated with 5 μL carboxylated FICT-labeled latex beads 2 μL in diameter (Sigma) for 90 min in darkness at 37 °C. Cells were washed, suspended in 1 mL of PBS, and analyzed in a flow cytometer (Facs Vantage; Becton–Dickinson, Palo Alto, CA, USA). Results were expressed as a percentage of cells positive for phagocytosis and mean channel fluorescence, which correlates with the number of particles phagocytosed by the cells. Tests were performed in triplicate and control assays were carried out at 4 °C, to eliminate the background fluorescence. Results were obtained as the percentage of cells positive for phagocytosis.

### 2.8. Statistical Analysis 

Mean comparisons were conducted by one-way ANOVA and Student–Newman–Keuls multiple comparisons. Significance was set at *p* < 0.05. Data were expressed as means ± standard deviation (SD) in all cases. Before ANOVA, normal distribution was probed by Kolmogorv–Smirnov tests (*p* > 0.05).

## 3. Results

### 3.1. Cell Proliferation Assay 

**Figure 1:**
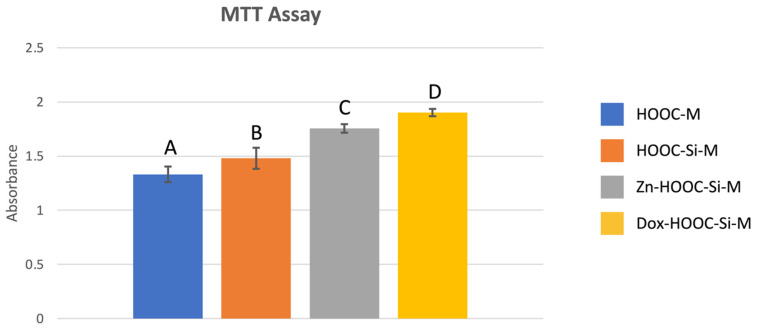
Absorbance mean values and standard deviations obtained after the MTT assay for the different membranes. Distinct letter indicates significant difference between membranes after ANOVA and Student–Newman–Keuls multiple comparisons (*p* < 0.05).

Mean and standard deviations of osteoblasts cell proliferation determined by the MTT assay are presented in Figure 1. Significant differences were found between all tested groups and attained means follow the trend: COOH-M < HOOC-Si-M < Zn-HOOC-Si-M < Dox-HOOC-Si-M. 

### 3.2. Field Emission Scanning Electron Microscopy (FESEM) 

**Figure 2:**
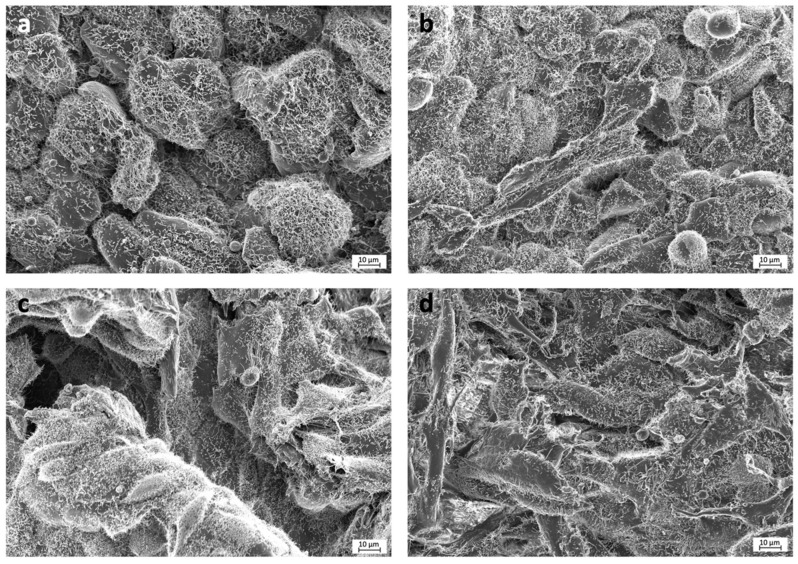
FESEM images of the experimental membranes (**a**) COOH-M, (**b**) COOH-Si-M, (**c**) Zn-COOH-Si, and (**d**) Dox-COOH-Si-M. Cells seeded on COOH-M are round shaped and filapodia are not observed. In the rest of the membranes, elongated cells are observed, some filapodia may also be detected emerging from osteoblasts cytoplasm. Osteoblasts are covered by fibrilar substance and rounded mineral deposits.

**Figure 3:**
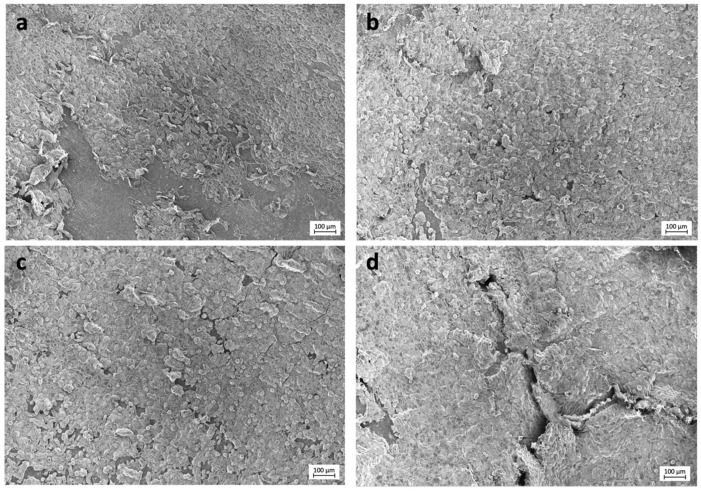
FESEM images of the experimental membranes (**a**) COOH-M, (**b**) COOH-Si-M, (**c**) Zn-COOH-Si-M, and (**d**) Dox-COOH-Si-M. Several aligned osteoblasts connected to each other may be seen. Thicker layers of osteoblasts, constituting a tri-dimensional structure, are evidenced on Zn-COOH-Si-M and Dox-COOH-Si-M.

Selected images from the FESEM analysis are displayed in Figure 2 and Figure 3. Osteoblasts cultured on COOH-Si-M displayed an elongated spindle-shaped morphology and a similar situation was observed for those grown on Zn-COOH-Si-M. By contrast, osteoblasts on COOH-M are round shaped. For osteoblasts on the Dox-COOH-Si-M the elongated morphology was accompanied by larger and more cytoplasmic membrane protrusions compared with those grown on the other membranes. There also exists an alignment of the elongated cells on the tested surfaces, which provided large bio-adhesive areas for the cells. Cell spreading and cell layer thickness is lower at COOH-M than in the other membranes. 

### 3.3. Alkaline Phosphatase (AP) Activity 

**Figure 4:**
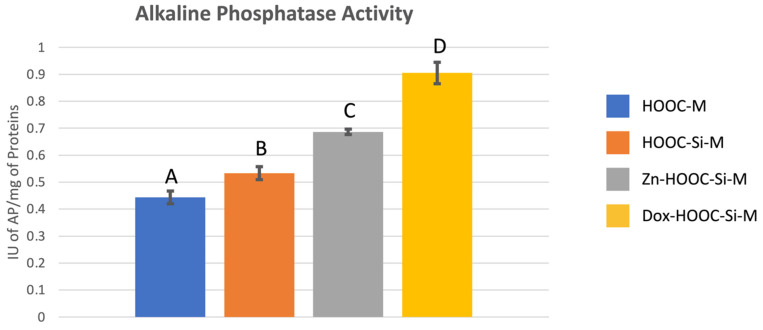
Mean and standard deviation of international units of AP per mg of proteins values obtained with the different membranes. Distinct letter indicates significant difference between membranes after ANOVA and Student–Newman–Keuls multiple comparisons (*p* < 0.05).

Mean and standard deviations of alkaline phosphatase expressed as international units (IU) of AP per mg of total proteins are presented in Figure 4. Significant differences were found between all tested groups and attained means follow the trend: COOH-M < HOOC-Si-M < Zn-HOOC-Si-M < Dox-HOOC-Si-M. 

### 3.4. Antigenic Phenotype 

**Figure 5:**
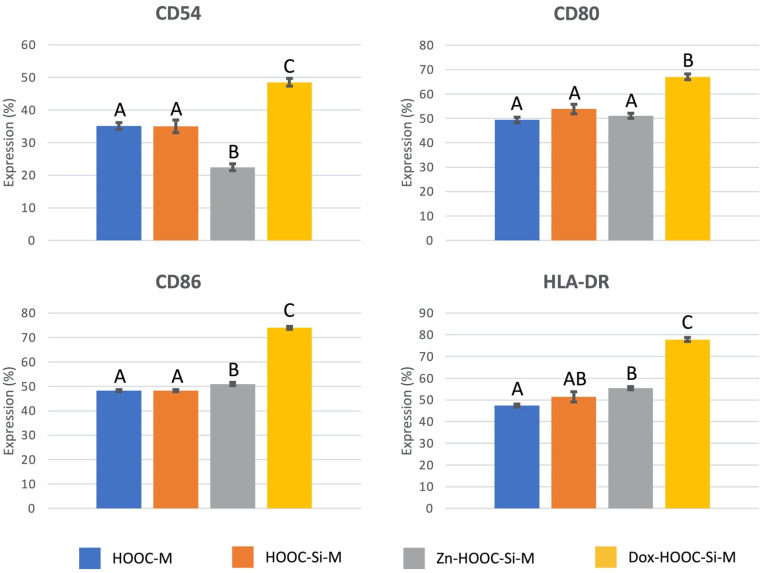
CD-marker antigenic expression on osteoblasts, cultured on the different experimental membranes. Values are presented as percentage of cells expressing the antigen phenotype (CD54, CD80, CD86, and HLA-DR). ANOVA and Student-Newman-Keuls multiple comparisons were performed (*p* < 0.05). Distinct letter indicates significant difference between membranes and always compares each CD-marker independently.

The characterization of cultured osteoblasts on the distinct membranes, for the presence of selected surface markers is presented in Figure 5. Osteoblasts cultured on doxycycline functionalized membranes presented higher expression of CD54, CD80, CD86, and HLA-DR (*p* < 0.05), than the rest of the groups. Differences were about (38%, 36%, 53%, and 64%, respectively). Zinc functionalization reduced CD54 expression on osteoblasts about 64% (*p* < 0.001).

### 3.5. Phagocytic Activity

Osteoblasts cultured on tested membranes were negative for phagocytosis, except for those osteoblasts grown on the COOH-M where 24.33% of cells were positive, and the standard deviation was 1.52%.

## 4. Discussion

This research manuscript implies an approach to the study of how osteoblasts’ proliferation and differentiation are affected by the different studied nanostructured polymeric membranes. MG-63 osteoblast-like cells were used for this in vitro study. They are one of the cell lines most widely used in literature since there are no interspecies differences with primary human osteoblasts, they have a shorter isolation time and there is unlimited accessibly [1,26]. However, the results need to be extrapolated with caution, taking into account that a tumor line may have an alternative pattern of differentiation from primary human osteoblasts [27,28]. The MTT assay and FESEM were used in order to quantitively measure osteoblasts’ proliferation while their differentiation potential was evaluated by means of alkaline phosphatase activity, antigenic phenotype expression, phagocytic activity, and FESEM. 

The previous surface characterization of the present membranes demonstrated that the mean fiber diameter was of around 765 nm [7]. Taking into account that mineralized collagen fibrils are about 800 nm in human trabecular bone, these membranes imitate the osseous structure. This collagen bone mimicking has been shown to increase cell attachment on membranes by about 1.7 fold [29]. The best strategy when designing an ideal scaffold is trying to replicate the native tissue and the fibrillar structure, enhancing cellular attachment, proliferation, and differentiation [1,7]. 

For topical drug application, the in vitro drug release assay does not correlate with the expected drug activity. In these cases, product performance tests in similar conditions to the clinical scenario have been employed. The present methodology involves the determination of the drug activity on osteoblastic cells, considering the liberated drug and the amount of drug permeated into the cells. The effect on cells of the quantity of drug which is not liberated but remained doing its action on the functionalized membrane is also considered. Moreover, it should also be taken into account that membranes are non-resorbable and do not swell or dissolve. Therefore, doxycycline and zinc release is controlled by diffusion of the drug/ions through the liquid permeating the membrane, but as membranes are bioactive [7], an apatite layer can be formed on the surface, hindering the diffusion of the drug/ions to the surrounding tissue [30].

Osteoblasts’ proliferation increased on silica-doped membranes, but the best results were obtained on doxycycline functionalized membranes (Figure 1). It has been previously reported that tetracyclines promote the proliferation of osteoblasts [4]; but, the underlying mechanism is not completely understood. Two different events may explain this fact: (i) osteoblasts’ proliferation is usually enhanced in the presence of antioxidants, as reactive oxidant species diminish osteoblasts’ reproduction [31], and doxycycline presents a potent antioxidant effect [32,33]. (ii) Doxycycline possesses calcium chelating ability that may facilitate calcium deposit on membranes [7]. Increases in extracellular calcium concentration have been shown to stimulate osteoblastic lineage cells, leading to bone formation and osteoconductivity [34]. 

Osteoblasts with an elongated morphology were observed on the doxycycline doped membranes, with filapodia extensions from the cells to the substrate, producing an interlocked cell structure (Figure 2). This suggests that interplay between the cell and the membrane surface allows for enhanced dynamic propagation and an overall increase in osteoblast activation, as indicated by the filopodia. Correlations between cell morphology and both proliferation and metabolic activity have been previously stated [1,35,36]. Hence, osteoblast spreading apparently favored the metabolic activity and cell elongation favored proliferation, while cellular roundness decreased mitotic activity [35,36]. The same correlation was found in the present study, obtaining the highest osteoblasts proliferation for the Dox-COOH-Si-M. Rounded and less spread cell shape was found on the COOH-M (Figure 2), coinciding with a lower cell proliferation. The described correlation of cell spreading and proliferation has already been reported, not only for osteoblasts but for other cell types [35]. The cellular morphology seems to be also affected by the presence of silica, inducing a three-dimensional growth (Figure 3). This three-dimensional cellular network is the basis for the known in vitro earlier differentiation of osteoblasts and better osseointegration in vivo [37].

Regarding osteoblasts differentiation, it may be ascertained from phosphatase activity assays that the silica blending and doxycycline doping of the membranes extremely induces osteoblasts’ differentiation since it increased the phosphatase activity in about 100% respect COOM-M (*p* < 0.001). The AP activity was also enhanced by 50% when osteoblasts were seeded on Zn-COOH-Si-M when compared with COOH-M (*p* < 0.001). It could be also ascertained that regarding AP activity, Dox and Zn could be said to favor osteoblasts’ differentiation, as doping COOH-Si-M with Dox and Zn, raises the AP activity by 70 and 30%, respectively (*p* < 0.001 in both cases). In previous investigations, the modulation of genes related to the osteogenic functional capacity of osteoblastic cells exerted by the studied membranes was evaluated. One of these genes was the one encoding the alkaline phosphatase, and the same tendency that the observed in the present study was registered [28]. 

It was found that osteoblasts expressed major histocompatibility complex molecules as HLA-DR, and adhesion molecules as CD54, as well as some signaling or co-stimulatory molecules as CD80 and CD86 [38]. The expression of these molecules is up-regulated by the tested doxycycline-doped membranes (Figure 5). Expression of HLA-DR, which has also been described on osteoblasts obtained after mandibular surgery [39], is related to osteoblasts’ osteocalcin production when stimulated with 1,25-di-hydroxyvitamin D3 [40]. The co-stimulatory molecules CD80 and CD86 are withal expressed by antigen presenting cells and play pivotal roles in inducing T-cell immunity or tolerance [41,42]. CD80 may primarily inhibit the immune response, working on T-lymphocytes [43].

The co-stimulatory molecule CD86 is a marker expressed in different cell populations such as dendritic cells. To understand the meaning of its over-expression it should be taken into account that the differentiation of osteoclasts is tightly regulated by bone-forming osteoblasts [44]. Osteoblasts express cytokines essential for osteoclastic differentiation, like the receptor activator of NF-kB ligand (RANKL) [45,46], in response to osteotropic hormones and factors [47]. Doxycycline and minocycline have been previously shown to induce the production of dendritic cell markers, like CD86, in the presence of RANKL [48]. Osteoclast’s precursors express RANK (RANKL receptors) and differentiate into osteoclasts in response to RANKL emitted by osteoblasts [46,47]. But tetracyclines convert the differentiation pathway, resulting in dendritic-like cells rather than osteoclasts, in the presence of RANKL in vitro and in vivo [48]. As a result, tetracyclines prevent bone loss [48], but the mechanism involved is only partially known. Further research is required in this field. Doxycycline and minocycline inhibited RANKL, which induced osteoclastogenesis, however, no other clear effect on cell growth and phagocytic activity of osteoclasts has been described. 

Zinc functionalization reduced CD54 expression on osteoblasts and doxycycline produced its over-expression (Figure 5). CD54 is an adhesion molecule and its presence on osteoblasts is well documented [39,49]. CD54 upregulation on osteoblastic cells has been shown to facilitate osteoclastogenesis [50,51]. CD54 is also known as intercellular adhesion molecule 1, a highly glycosylated immunoglobulin superfamily member that binds the leukocyte integrins. CD54 is constitutively expressed on leukocytes, epithelial, endothelial, and other cells, and it is up-regulated in response to inflammatory mediators [38,52]. CD54 is the mediating protein involved in the contact between mesenchymal stromal cells and macrophages. It has an essential role in regulating immunosuppressive properties of mesenchymal stromal cells [53]. When CD54 is over-expressed by cells, they become more immunosuppressive in inflammatory environments [54]. There is an unconventional but functional CD54-mediated interaction between pro-inflammatory macrophages (M1) and mesenquimal cells. This crosstalk modulates the immunosuppressive functions of mesenquimal cells and opens important perspectives in therapies for autoimmune and inflammatory diseases [53].

Doxycycline has previously been described as a potent anti-inflammatory and antioxidant substance [33]. These properties are related to beneficial doxycycline actions stated on alveolar tissue inflammatory infiltrates [13]. Oral diseases producing bone loss, such as periodontitis or periimplantitis, are characterized by inflammation and produce the activation of immunological cells leading to the release of pro-inflammatory cytokines and the recruitment of phagocytes and lymphocytes [55]. 

Phagocytosis is a biological cellular activity through which cells can protect themselves from infectious and non-infectious environmental particles. The process of phagocytosis has been previously identified in osteoblasts [25,56] and requires the recognition of ligands by specific receptors expressed by the phagocyte. Receptor engagement triggers intracellular signaling pathways that initiate appropriate innate immune and pro-inflammatory responses [57]. Osteoblasts grown on tested functionalized membranes possess a diminished phagocytic activity, as an additional sign of anti-inflammatory response to tested doped biomaterials. Just the COOH-M presented about 25% of osteoblasts positive to phagocytosis activity. 

Biomaterial-mediated inflammatory response is crucial for bone regeneration. Excessive inflammation may lead to the formation of fibrous tissue, preventing bone cells from integrating with the membranes, resulting in the failure of bone regeneration. However, moderate inflammatory responses may enhance the recruitment and differentiation of osteoblasts, improving osteogenesis [58]. Further research with macrophages in co-culture is required determining immunomodulation and osteogenic differentiation. 

Despite recent advancements, the therapeutic capability of biomaterials to regulate the osteoblastic cells-host immune system crosstalk, and the mechanism underlying this immunoregulation is poorly understood [14]. The most important limitation of the present study is the lack of mechanistic assays. When the application of detailed mechanistic assays is being intended [31], it may help to gain insight into a particular mechanism of action, but it also hinders the multi-regulatory processes that are necessary in the biological complexity. However, the efficient use of methods, enabling the identification of cells antigenic phenotype, has an enhanced probability of success in translation of new biomaterials through to the clinic. It presents a different perspective on signaling focused on integrated or holistic responses rather than the resolution of individual events. However, it is recognized that further screening efforts are necessary in order to discover an array that really allows the study and probing of detailed mechanistic studies, for example how these receptors are activated/inactivated and then to ascertain the dissection of cellular pathways. Rather than simplifying, the increasing number of tools should contribute to refine models and to cover further levels of biological complexity. Analogous to a puzzle built from multiple individual pieces, the full representation of cellular activity may transcend following the assembly of different functional outputs. 

Since membrane exposure to the oral cavity and contamination of the surgical site are common problems, the linking of antimicrobial [9], anti-inflammatory, and bone regeneration property of doxycycline functionalized membrane makes it a favorable alternative therapeutic tool for GBR procedures prior to implant placement.

## 5. Conclusions

In the present study, it has been demonstrated that silica loading may offer beneficial effects to experimental membranes in terms of osteogenicity (osteoblasts proliferation and differentiation). Adding zinc to the membranes’ formulation showed an enhancement in the proliferation capacity of osteoblast. Furthermore, even better results were obtained when the scaffolds were functionalized with doxycycline. An up-regulation of several antigenic markers with immune-modulatory potential has also been demonstrated for these Dox-COOH-Si-Ms, which may be potential candidates for use in GBR procedures in several challenging pathologies, including periodontal disease.

## Data Availability

The data presented in this study are available on request from the corresponding author.
